# SGF-SLAM: Semantic Gaussian Filtering SLAM for Urban Road Environments

**DOI:** 10.3390/s25123602

**Published:** 2025-06-07

**Authors:** Zhongliang Deng, Runmin Wang

**Affiliations:** 1 School of Electrical and Information Engineering, Zhengzhou University, Zhengzhou 450001, China; dengzhl@bupt.edu.cn; 2 School of Electronic Engineering, Beijing University of Posts and Telecommunications, Beiing 100876, China

**Keywords:** mapping, SLAM, vision-based navigation, road environment

## Abstract

With the growing deployment of autonomous driving and unmanned systems in road environments, efficiently and accurately performing environmental perception and map construction has become a significant challenge for SLAM systems. In this paper, we propose an innovative SLAM framework comprising a frontend tracking network called SGF-net and a backend filtering mechanism, namely Semantic Gaussian Filter. This framework effectively suppresses dynamic objects by integrating feature point detection and semantic segmentation networks, filtering out Gaussian point clouds that degrade mapping quality, thus enhancing system performance in complex outdoor scenarios. The inference speed of SGF-net has been improved by over 23% compared to non-fused networks. Specifically, we introduce SGF-SLAM (Semantic Gaussian Filter SLAM), a dynamic mapping framework that shields dynamic objects undergoing temporal changes through multi-view geometry and semantic segmentation, ensuring both accuracy and stability in mapping results. Compared with existing methods, our approach can efficiently eliminate pedestrians and vehicles on the street, restoring an unobstructed road environment. Furthermore, we present a map update function, which is aimed at updating areas occluded by dynamic objects by using semantic information. Experiments demonstrate that the proposed method significantly enhances the reliability and adaptability of SLAM systems in road environments.

## 1. Introduction

Currently, traditional SLAM techniques excel in tracking, but they fall short of meeting the demands for high-quality mapping [1]. Although 3D Gaussian splitting [2] has gained significant attention due to its ability to produce high-quality mapping results, it heavily relies on accurate point cloud positional relationships and thus depends on robust 3D reconstruction techniques, such as SFM (structure-from-motion), during the initialization phase. Traditional SFM methods, such as COLMAP [3], are highly effective for object reconstruction but struggle in complex open scenes and road environments, especially those lacking loops or sufficient viewpoints. For instance, road datasets like KITTI360 [4] and KITTI [5] cannot achieve accurate initialization with COLMAP due to the mismatch between the scene characteristics of these datasets and COLMAP’s object-centric reconstruction approach.

To tackle this challenge, recent studies have sought to integrate SLAM technology with Gaussian splatting [6,7], leveraging SLAM’s localization capabilities to enable high-quality scene reconstruction and provide accurate localization services. These integrated approaches have seen notable success, particularly in indoor environments and some synthetic datasets [8,9]. Methods such as SplaTAM [10] and GS-SLAM [6] combine 3D Gaussian splatting with SLAM, leveraging its realistic scene reconstruction capabilities to outperform NeRF-based [11] SLAM methods in rendering quality. RGBD GS-SLAM [12] introduced a dense visual SLAM system based on 3D Gaussian splatting, using the Generalized Iterative Closest Point algorithm for pose estimation, which significantly enhanced the system’s tracking accuracy and processing speed. MonoGS [7] implemented the first fully 3DGS-based monocular SLAM system, supporting monocular and RGB-D inputs, and demonstrated advanced tracking and mapping performance across multiple datasets. Photo-SLAM [13] integrates ORB-SLAM3 with Gaussian splatting technology, improving tracking and mapping performance in Extensive datasets. DroidSplat [14] is capable of implementing an end-to-end SLAM system without known camera intrinsics, incorporating the latest 3DGS renderer. Compact-SLAM [15] proposed a compact 3DGS SLAM system that reduces the number and parameter size of Gaussian ellipsoids. Among these open-source methods, only Photo-SLAM achieves tracking accuracy comparable to traditional SLAM systems. However, most SLAM methods based on 3D Gaussian splatting are primarily limited to indoor RGB-D datasets, with insufficient research addressing monocular or stereo camera in outdoor road environments [16]. Our testing shows that in complex and dynamic scenes, the performance of those indoor methods significantly declines, especially in outdoor environments, where inadequate frontend localization ability remains the key limiting factor.

In outdoor traffic-road environments, significant advancements have also been made in the application of 3DGS technology [17]. Some methods specifically designed for road environments, such as CityGaussian [18] and VastGaussian [19], have also struggled to overcome this fundamental limitation. CityGaussianV2 [20] introduced a 3D Gaussian splatting-based method for road scene modeling that effectively handles dynamic objects, delivering high-quality mapping results. VastGaussian [19] proposed a dense visual SLAM system based on 3DGS, which achieves high-quality mapping and localization in large-scale outdoor environments. Street Gaussians [21] introduced a dynamic street modeling method using 3DGS, effectively processing dynamic objects. The HUGS [22] method combines static and dynamic 3D Gaussians to jointly optimize geometry, appearance, semantics, and motion, with the posture of moving objects regularized through physical constraints. This approach enables real-time rendering of new viewpoints and reconstruction of dynamic scenes. S3Gaussian [23] proposed a self-supervised dynamic scene modeling method based on 3DGS, achieving high-quality mapping and localization for autonomous driving scenarios. These research findings highlight the broad application and potential of Gaussian splatting technology in outdoor environment modeling. However, these methods do not achieve real-time tracking and lack the full functionality of SLAM, which poses a significant challenge in integrating SLAM with Gaussian splatting in street environments. Most of these methods rely on SFM techniques for initialization, which cost tons of time to initialize and have shown poor performance in real-world experiments. In addition, despite those non-SLAM approaches that introduce more sophisticated mapping mechanisms, the core issue, localization accuracy, remains unresolved and costs tons of time to initialize by SFM.

In addition to pure visual solutions, LiDAR-fused Gaussian mapping methods significantly enhance geometric accuracy and robustness in dynamic and large-scale scenarios by integrating LiDAR data with Gaussian mapping techniques. LiV-GS [24] introduces conditional Gaussian constraints to achieve efficient cross-modal alignment and novel view synthesis; LiGSM [25] leverages LiDAR to initialize Gaussian mappings and combines joint supervision from images and depth maps to improve the accuracy of pose estimation and scene rendering; LI-GS [26] employs a planar-constrained multi-modal Gaussian mixture model to provide continuous supervision during initialization and optimization, significantly enhancing the precision of large-scale reconstruction. However, these methods still face challenges in handling dynamic occlusions and real-time performance, and their costs increase substantially with the addition of laser sensors compared to visual solutions. In contrast, DUSt3R [27] uses a Transformer architecture to regress dense point clouds directly from image pairs, eliminating the dependence on camera intrinsic parameters and poses, making it more adaptable, especially in unstructured and dynamic environments. However, it may be inferior to LiDAR-fused methods in geometric accuracy and global consistency. Moreover, these methods lack real-time performance.

Overall, SLAM strategies that prioritize tracking over mapping struggle to adapt to complex outdoor scenarios. To address these challenges above, we focused on integrating ORB-SLAM3 [28], one of the most robust SLAM systems, with Gaussian splatting technology named Photo-SLAM [13] and found that it achieves the highest success rate in both tracking and mapping across various datasets. However, despite its strong localization performance, Photo-SLAM suffers from suboptimal mapping quality, and its rendering capability is significantly compromised by dynamic environmental changes in complex road scenarios. Moreover, handling dynamic changes in road environments to produce cleaner, more accurate maps, while improving localization in these challenging conditions, remains a critical issue.

To address this issue, this paper proposes a novel approach that integrates semantic information, effectively combining traditional SLAM technology with Gaussian splatting. We design a frontend system that incorporates both feature point detection and semantic segmentation. By utilizing semantic information throughout the entire localization and mapping process, we are able to filter out redundant data and reduce the impact of dynamic objects. This procedure is referred to as Gaussian point cloud filtering. In this system, semantic information is used not only to guide the selection of feature points but also to influence the generation of Gaussian initialization. Specifically, we apply occlusion masking to dynamic objects (such as pedestrians and vehicles) in road environments, preventing these objects from negatively affecting the mapping process. To improve frontend processing efficiency, we incorporate a good performance lightweight STDC-seg [29] semantic segmentation module and integrate it with our feature point matching module. Our method has been experimentally validated in various environments, yielding promising results. The method includes three key innovations:

- We designed and modified a new multi-task frontend network, integrating semantic segmentation and feature point detection tasks, and trained the network with an appropriate loss function, achieving both fast and accurate performance.
- We proposed a Semantic Gaussian Filter technique for street environments, designing a dynamic mapping framework that effectively shields dynamic objects that change over time in the scene.
- We combined semantic information to continuously update the map for dynamic occlusion areas. This provides stable navigation services and map rendering capabilities.

The rest of this paper is organized as follows. Section 2 presents a theoretical analysis and descriptions of different algorithm parts of SGF-SLAM, including multi-task network, Semantic Gaussian Filter algorithm, and map reload and update module. The simulation and real-world experiment results are provided in Section 3, followed by the discussion in Section 4. We conclude this work in Section 5.

## 2. Materials and Methods

**Figure 1:**
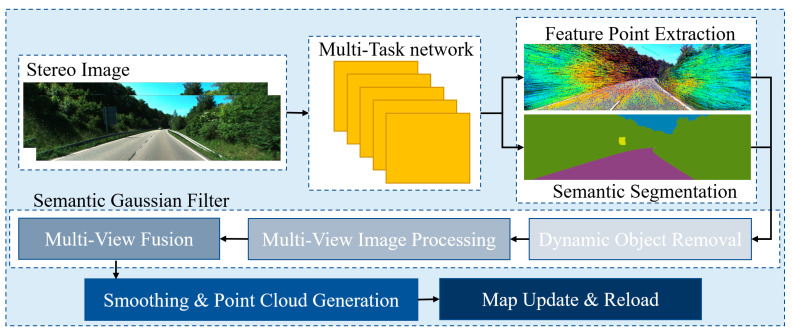
The architecture of SGF-SLAM.

The architecture of SGF-SLAM is shown in Figure 1. Our method is based on Photo-SLAM [13], with multiple innovations. We created a multi-task network that is used in both tracking and mapping processes. We have developed a Semantic Gaussian Filter method called SGF. It includes dynamic object removal, multi-view image processing, and multi-view fusion, which effectively removes dynamic objects such as pedestrians and vehicles during the Mapping process. At the same time, our network provides stable tracking performance, allowing our SLAM method to achieve optimal performance in multiple aspects.

### 2.1. Multi-Task Network

To enhance the performance of SLAM systems by integrating semantic information, we propose a scheme called SGF-net that combines a semantic segmentation network with a feature point detection network. Our goal is to design an efficient and accurate frontend network that supports both tracking and mapping, where extremely high segmentation accuracy is not a strict requirement. This design allows us to maintain a lightweight frontend semantic detection network.

**Figure 2:**
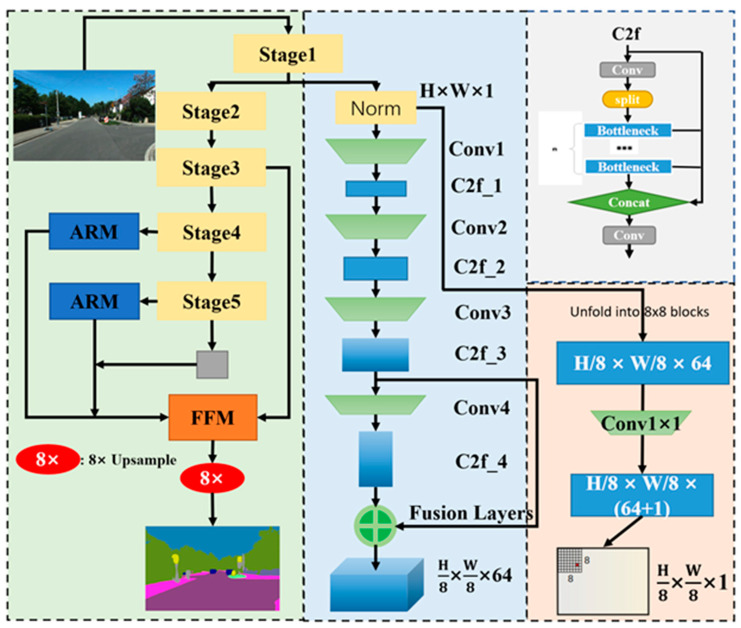
The architecture of SGF-net.

Our network is an efficient multi-task network that outputs three components from a single image. These components include a semantic segmentation map of the same size as the original image, a heatmap of size H/8 × W/8 × 64, and the corresponding descriptor. As shown in Figure 2, we select the fast and efficient STDC-seg network [29] as the core framework for semantic segmentation. Based on a CNN structure, this network strikes a good balance between speed and accuracy. For feature point detection, we utilize the Cross-layer Feature Fusion module (C2F) from YOLOv8 [30] as the foundational block of the detection network. This module consists of multiple convolutional layers and bottleneck structures that are combined to optimize performance. The C2F module has been shown to significantly reduce computational complexity while retaining the ability to capture spatial information effectively. Its integration enables our network to achieve efficient data compression and information transfer during feature extraction, ensuring low computational cost and high inference speed. Additionally, the feature point detection network and semantic segmentation network share a common head network. The design of the SGF-net fusion module aims to achieve efficient unification of feature extraction and semantic segmentation through a lightweight feature fusion strategy. Compared with the complex multi-task coupling architectures in SLAM and the complex processing flows of semantic-focused networks, its structure is more concise, avoiding the redundancy of multi-module stacking. Through the deep collaboration of task-specific modules, it forms a tighter task coupling relationship, rather than the loose association through late-stage integration, thus demonstrating advantages in structural efficiency, inference speed, and tasks cooperation. This architecture strikes a balance between real-time performance and the effective integration of semantic information for dynamic object suppression, ultimately enhancing the robustness and mapping quality of the overall SLAM system.

### 2.2. Network Loss

For the semantic component, we follow the training methods outlined in [29]. The feature point detection network will output a heatmap and the corresponding descriptor. The training loss function for the heatmap consists of two parts: the standard detection loss [31] and a seg loss based on semantic segmentation results. The core idea behind the seg loss is to encourage feature points to be concentrated in regions with stable objects, thereby reducing the impact of dynamic objects and background noise. In this work, we define pedestrians and cars as unstable objects. For the descriptor loss, we adopt a loss strategy similar to that used in SuperPoint [32].

The heatmap detection loss uses Hamming distance to measure the matching accuracy between the detected feature points and the ground truth feature points. This loss term incentivizes the model to detect as many feature points as possible. We use the detection results from Xfeat network [31], with a threshold of 0.13 and NMS set to 5, as the ground truth.

(1) $$L_{feat}=\sum_{i}\left(∥p_{i}-p_{i}^{gt}{∥}^{2}\right)$$

$p_{i}$ represents the predicted feature point, while $p_{i}^{gt}$ denotes the ground truth feature point. To enable the model to exclude feature points located in dynamic object areas during training, we introduce a loss term specifically designed to penalize feature points detected on dynamic objects:

(2) $$L_{dynamic}=\sum_{i\in P_{dynamic}}I\left(p_{i}\in P_{dynamic}\right)\cdot \lambda_{dynamic}∥p_{i}-p_{i}^{gt}{∥}^{2}$$

$P_{dynamic}$ represents the area containing feature points from dynamic objects, and I is the indicator function that identifies whether feature points belong to dynamic objects, where they should be ignored. $\lambda_{dynamic}$ is a hyperparameter that controls the intensity of the penalty applied to feature points in dynamic object areas, with a default value of 0.01. This loss term encourages the model to minimize the number of feature points generated within dynamic object regions. To further guide the model toward generating feature points on stable objects, we introduce a reward loss that incentivizes the model to focus on static objects:

(3) $$L_{stable}=\sum_{i\in P_{stablc}}I\left(p_{i}\in P_{stable}\right)\cdot \lambda_{stable}∥p_{i}-p_{i}^{gt}{∥}^{2}$$

$\lambda_{stable}$ is the hyperparameter that controls the reward for generating feature points on stable objects, with a default value of 0.02, and $P_{stable}$ represents the area of the stable object. The final loss function can be expressed as

(4) $$L_{total}=L_{feat}+\lambda_{1}L_{dynamic}-\lambda_{2}L_{stable}$$

$\lambda_{1}$ and $\lambda_{2}$ are hyperparameters, set to default values of 0.12 and 0.08, respectively, and are used to balance the intensity of penalties for dynamic objects and rewards for stable objects.

### 2.3. Semantic Gaussian Filter

This section presents the dynamic mapping method based on semantic masking of dynamic objects and multi-view geometry. During the feature point detection process, we leverage the semantic information provided by the semantic segmentation network to filter out feature points located in dynamic object areas and exclude these areas from the mapping process. Let S(x) denote the semantic label of a pixel x in the image. The pixel will be ignored during feature point detection if S(x) corresponds to a dynamic object. Specifically, for each pixel x in the image, we apply a mask to make the following determinations:

(5) $$\left.mask(x)=\left\{\begin{array}{ll} 0 & ifS(x)\in \{car,pedestrian\}\\ 1 & otherwise \end{array}\right.\right.$$

Along with removing dynamic objects, the appearance of occluded areas must also be addressed. Dynamic objects can cause occlusion, leading to void areas during feature point detection, where effective feature point information is missing. To recover these void areas, we apply multi-view geometry to estimate the appearance of the occluded regions by leveraging the co-visual relationships between multiple perspectives.

**Figure 3:**
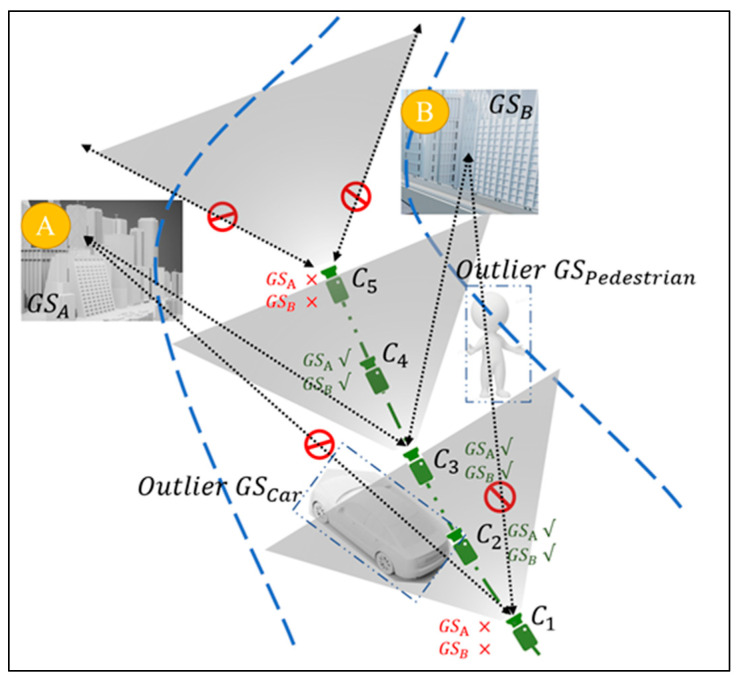
Schematic diagram of multi-view geometry in road environments.

The schematic diagram of multi-view geometry in road environments is shown in Figure 3; C indicates that the camera has different viewing angles, where $C_{1}$, $C_{2}$ and $C_{5}$ cannot see the full appearance of $GS_{A}$ and $GS_{B}$, but $C_{3}$ and $C_{4}$ have no obstruction of the viewing angle. When occlusion in $C_{1}$ and $C_{2}$ is recognized, they do not generate the corresponding Gaussian in that area, so that when $C_{3}$ and $C_{4}$ generate Gaussian points in the corresponding area, the occluded angle of view can still be restored. The opacity and color of the Gaussian point are continuously optimized in iterations. The mathematical model is as follows.

We suppose that in the current view $C_{i}$, an area lacks feature points due to occlusion by dynamic objects. We can use images from other viewpoints, such as $C_{j}$, to fill in this gap. Using the camera pose information, we can compute the co-visual relationship between the current viewpoint and other viewpoints, allowing us to estimate the geometry of the occluded area. Let the relative pose between the current viewpoint $C_{i}$ and viewpoint $C_{j}$ be denoted as $T_{i,j}$, and the occluded area in the common view as $R_{i_{j}}$. The recovered area $R_{i}$ can then be represented as follows:

(6) $$R_{i}=R_{i}\cup \underset{j=1}{\overset{N}{⋃}}T_{i,j}\left(R_{i_{j}}\right)$$

Let N represent the number of perspectives. $T_{i,j}\left(R_{i_{j}}\right)$ denotes the projection of the recovered area $R_{i_{j}}$ from viewpoint $C_{j}$ onto the current viewpoint $C_{i}$. In this way, the occluded area is effectively restored, and mapping errors caused by occlusion are avoided.

Although dynamic objects have been removed and the occluded areas restored using semantic information, some regions of the image may still produce unstable point clouds due to residual effects of dynamic objects. These unstable regions can interfere with the final mapping results. To further refine the mapping results, we introduce a smoothing filter method to process images that have undergone semantic filtering. The goal of the smoothing filter is to generate regions with colors similar to the surrounding environment and avoid generating Gaussian point clouds in these regions. Specifically, for each image frame, dynamic object regions are first identified using a semantic segmentation network. Then, a smoothing filter is applied to these regions so that their colors blend with the surrounding environment, thereby reducing the generation of Gaussian point clouds caused by dynamic objects.

The smoothing filter is implemented as follows: let the color of a region in the image be C(x) and the color of its surrounding area be $C_{neighbors}(x)$. We use the following filter for smoothing

(7) $$C_{smooth}\left(x\right)=\alpha C_{neighbors}\left(x\right)+(1-\alpha )C(x)$$

where α is the smoothing coefficient that controls the degree of smoothing. For the smoothed region, we skip it during Gaussian point cloud generation. That is, if the smoothed color of a region is similar to its surroundings, no Gaussian point cloud is generated for that area. This process effectively avoids the influence of dynamic objects on the point cloud, making the final point cloud cleaner and more stable. Finally, the Gaussian point cloud P generated from the smoothed image can be represented as

(8) $$P_{final}=\{p_{i}|C_{smooth}(x_{i})\neq C_{smooth}(x_{dynamic})\}$$

where $p_{i}$ is each point in the Gaussian point cloud, $x_{i}$ is the pixel location of the point, and $C_{smooth}(x_{dynamic})$ is the color of the dynamic object region. This reduces obtrusive imaging effects, such as black holes, and minimizes blurring. Additionally, no Gaussian initialization points are generated in these regions of this frame.

### 2.4. Map Update and Reload

If a loop closure is detected, we stop the mapping process to avoid drastic degradation of the rendering due to odometry errors or shadow changes. However, we selectively update parts of the map. We have expanded the semantic information of dynamic areas. Specifically, we filter out dynamic regions that are too small and appropriately expand the regions of large dynamic objects to ensure that the area surrounding these objects is effectively shielded, particularly the shadows cast by the objects. Let the dynamic object region output by the semantic segmentation network be denoted as $R_{dynamic}$. We then expand this region to generate an extended region, $R_{extended}$, as defined by the following formula:

(9) $$R_{extended}=R_{dynamic}⊕B(\delta )$$

The symbol ⊕ represents the region expansion operation, and B(δ) denotes a structural element that controls the size of the expanded area, with δ being the expansion coefficient. We use a square region as the shape for the expanded area, and we typically set δ to 1.3 times the maximum lateral or vertical direction of the dynamic region. This will create a broader area around the dynamic object, helping to avoid misjudgments. The purpose of marking the area is to address the cavities and instability that may remain after the removal of dynamic objects. The premise is that there are no more dynamic objects here. We mark dynamic objects and their influence areas as key regions for map updates. These marked regions will be re-evaluated and updated during the Gaussian-rendered map update process. When the region is updated, the update mark will be cleared. This process will only be repeated once.

Map updates are essential for managing dynamic object occlusion and changes in the environment. With the removal of dynamic objects, the updated map can more accurately reflect the current state of the environment. Especially during long-term operation, environmental changes may cause feature points in certain areas to fail or become unstable. In such cases, we continuously update the map by combining semantic information and feature point matching.

**Figure 4:**
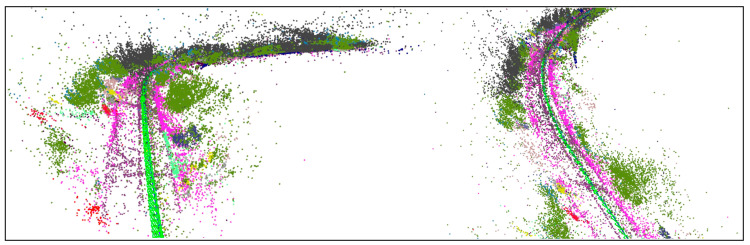
Reloadable semantic maps in KITTI360 dataset sequence 07 [4].

In traditional SLAM systems, feature point detection and matching are the most fundamental steps. However, factors such as dynamic environments and lighting changes can reduce the stability and matching accuracy of feature points. To enhance the robustness of feature points and the adaptability of the system, we employ the feature point detection network proposed in Section 2.1. This network can generate corresponding feature maps during each run and supports map reloading. The feature maps are shown in Figure 4. We have developed a reloadable semantic map by integrating semantic segmentation with an efficient feature point detection network.

During the map reloading process, we use feature point matching and pose estimation to match the newly generated feature points with those in the existing map. Based on the matching results, we estimate the current frame’s relative pose and update the map accordingly. The reloading process is expressed as

(10) $$T_{current}=M(P_{current},P_{previous})$$

where $T_{current}$ is the pose of the current frame, $P_{current}$ and $P_{previous}$ represent the feature point sets of the current and previous frames, respectively, and M(⋅,⋅) is the function for feature point matching and pose estimation. In this way, we can reload the map across different times and lighting conditions and provide stable navigation services for robots or autonomous driving systems.

## 3. Results

### 3.1. Setup

This section presents the results of our experiments across various datasets, including feature point detection models, odometry accuracy, mapping performance, and system evaluation. All experiments were conducted on the following hardware platform: Intel i5-13,600 KF CPU (Santa Clara, CA, USA), 32 GB RAM, NVIDIA RTX 3090 Ti (Santa Clara, CA, USA), and 3 TB of storage. The deep learning network pipeline is implemented using PyTorch, and the SLAM system is an enhancement of Photo-SLAM, divided into two components: the SLAM system and the map reload system, both of which are developed in C++. The network model is processed using TensorRT-10.2.0.19. During the densify and prune operation, we set the maximum gradient threshold to 0.0002 and the minimum opacity threshold to 0.65 for the Gaussians. In subsequent comparison experiments, PSNR, SSIM, and LPIPS indicators are calculated after excluding dynamic objects (vehicles and pedestrians).

### 3.2. Evaluation of Frontend Network

We trained the network on the KITTI360 dataset [4] and the Cityscapes dataset [33]. The resolutions in the KITTI dataset are 1242 × 375, the Cityscapes dataset has a resolution of 1024 × 2048. To validate the capabilities of our proposed feature point detection model, we conducted extensive testing using the HPatch dataset [34], in which the resolution of the RGB image is 640 × 480. The dataset contains a series of image pairs that have undergone different transformations, such as viewing angle changes, scale changes, and lighting changes. We compared the ORB [35], SuperPoint [32], and XFeat [31] alongside STDC-seg [29]. Since our network shares the same structure as STDC-seg in the semantic part, and the semantic segmentation results are similar, we will focus on testing the overall network performance and the feature point detection module. We also compared splitting the multi-task network into segmentation and feature detection network and training them separately to see how the combined networks improved speed; we refer to this approach as separate ours.

**Figure 5:**
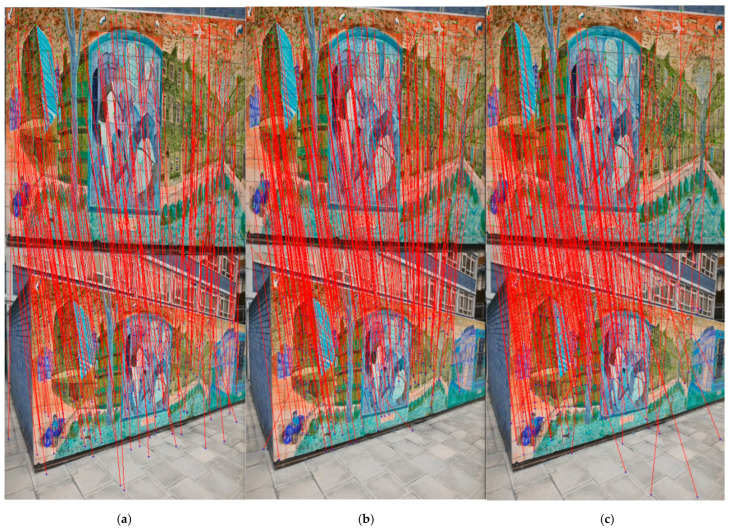
Qualitative analysis of feature matching results. (**a**) is Superpoint [32], (**b**) is SGF-net, and (**c**) is Xfeat [31].

The test results show that the proposed feature point detection network can stably extract feature points under varying lighting conditions, viewpoints, and dynamic environments, with strong interference resistance. Additionally, our network offers advantages in terms of speed, improving FPS by about 10 percent, compared to the separate network. Table 1 shows the quantitative analysis, and Figure 5 shows the qualitative analysis of the different networks.

### 3.3. Experiments on Odometer Accuracy

**Figure 6:**
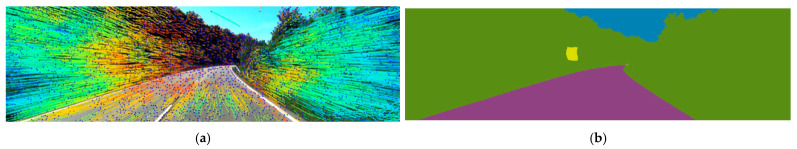
The results of feature point tracking and semantic segmentation of SGF-net in KITTI360 dataset sequence 07 [4].

In the odometer accuracy test, we used the KITTI Odemetry [37] datasets. The resolution of the RGB image is 1242 × 375. We reported the absolute translation RMSE $t_{abs}$ proposed in [38], and the average relative translation $t_{rel}$ and rotation $r_{rel}$ errors proposed in [37] from sequence 00 to 10. We compare the proposed method with Photo-SLAM [13], ORB-SLAM3 [28], and DROID-Splat [14] methods to evaluate the pose estimation accuracy under different conditions, such as dynamic object interference and complex scenes. Our method supports both stereo and monocular camera, while RGBD is not supported in street environments. Table 2 shows the quantitative analysis of the stereo camera. Figure 6 shows a qualitative representation of GSF-net’s tracking and semantic segmentation on a dataset. Experimental results show that, based on our feature point detection and dynamic object removal methods, the SLAM system can provide high accuracy and stability in complex environments. Especially in the presence of vehicles and pedestrians, our system is able to effectively reduce errors caused by dynamic objects, thereby improving the accuracy of the odometer.

### 3.4. Experiments on Rendering

**Figure 7:**
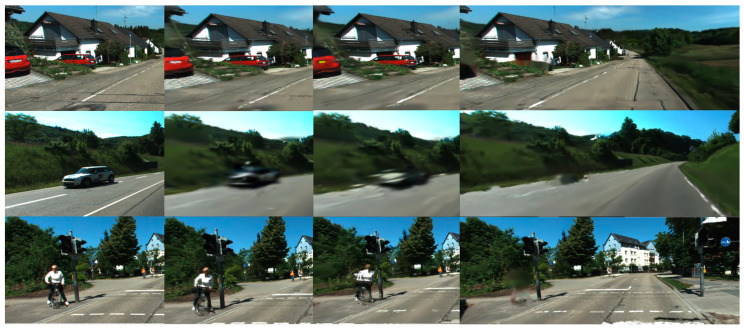
Qualitative comparison on the KITTI360 dataset. From left to right: ground truth, Hugs [22], Photo-SLAM [13], and Ours. The dataset sequences are 05, 07, and 10. Our method is SOTA in terms of dynamic object suppression, and our method performs well in rendering the details of the surrounding trees.

To evaluate the mapping effect of our proposal, we used the Virtual KITTI 2 [39] and KITTI 360 [4] road datasets. The resolution of the RGB image is 1242 × 375. To assess rendering quality, we report standard photometric rendering quality metrics (PSNR, SSIM, and LPIPS). We compared Photo-SLAM [13] and HUGs [22]. Table 3 shows the quantitative analysis, and Figure 7 shows the qualitative analysis of the different methods. In this experiment, we test the mapping effect of erasing vehicles and pedestrians, and the results show that the system can effectively remove the influence of dynamic objects and restore the occluded area by combining semantic information and feature point detection network for dynamic object removal. The accuracy of mapping is improved, and the quality of rendering is better than other methods in specific scenarios.

### 3.5. Experiments on Campus Datasets

**Figure 8:**
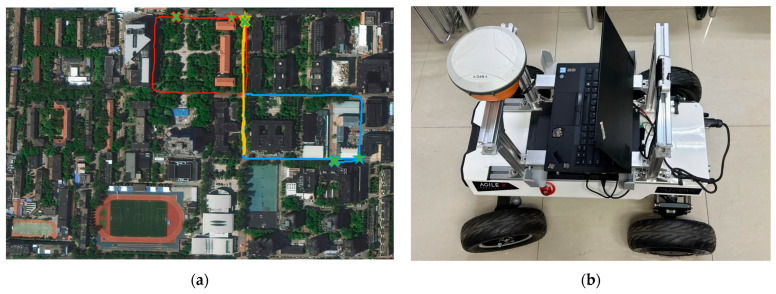
(**a**) Experimental trajectories of the campus dataset, where the star represents the starting point and the X represents the end point. Different colors represent different tracks; (**b**) experimental equipment.

In addition to the public dataset, we also conducted a series of tracking and mapping experiments in specific areas of Beijing University of Posts and Telecommunications. These areas include different types of roads, buildings, and a wealth of environmental features, and this is an important scenario for verifying the performance of our method in real-world applications. We mainly made comprehensive comparisons with Photo-SLAM [13] and Hugs [22] methods in average parameters such as PSNR, SSIM, LPIPS, RMSE, $t_{abs}$ [38], tracking FPS, and rendering FPS. We conducted multiple experiments around the west side of the Fourth Teaching Building and the Research Building on the BUPT campus, and Figure 8 shows the trajectory around the experiments and the measuring equipment. We use the realsense D435i camera to record datasets, and the Huayi e91 RTK receiver as the ground truth. The resolution of the RGB image is 1920 × 1080.

**Figure 9:**
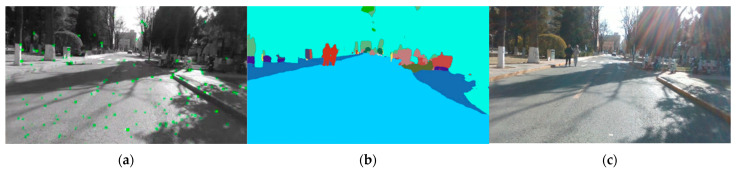
Performance of SGF-SLAM in real-world tests. (**a**) Feature point detection; (**b**) semantic segmentation; (**c**) RGB images.

**Figure 10:**
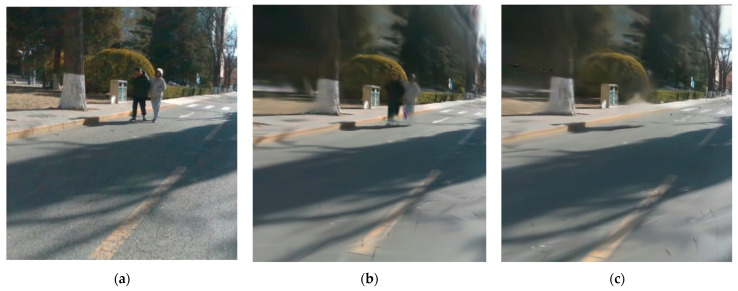
Qualitative analysis of BUPT campus datasets. (**a**) Truth Ground; (**b**) Photo-SLAM [13]; (**c**) ours. SGF-SLAM is effective in eliminating pedestrians.

Due to camera limitations, we can only render RGB images using a monocular camera. Therefore, we measured the monocular performance, which, together with the stereo performance from Experiment C, forms a complete experiment for both monocular and stereo camera. Table 4 shows the quantitative analysis of our experiments. Figure 9 shows the performance of SGF-SLAM in real-world tests. Figure 10 illustrates the qualitative analysis of the experimental results for BUPT campus scenario. Experimental results show that our method can achieve efficient and accurate road positioning and mapping in complex environments, especially in scenes with dense dynamic objects, and can maintain high positioning accuracy and map quality.

### 3.6. Experiments on Map Update and Reload

**Figure 11:**
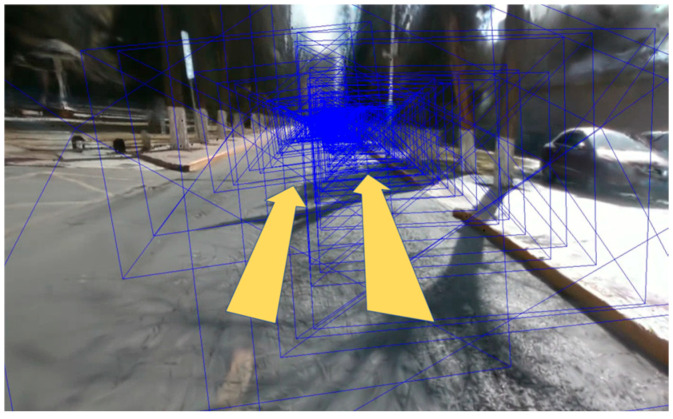
The comparison of the two trajectories after the loop showing the process of the map update function.

**Figure 12:**
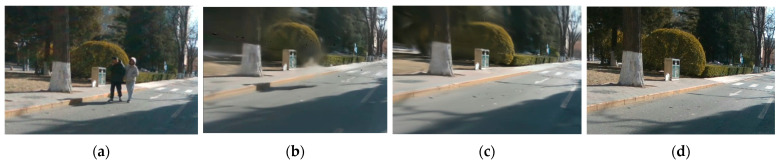
Qualitative analysis of map updates: (**a**) truth ground with pedestrians; (**b**) before map update; (**c**) after map update; (**d**) truth ground without pedestrians. The map was successfully updated when the loop occurred.

**Figure 13:**
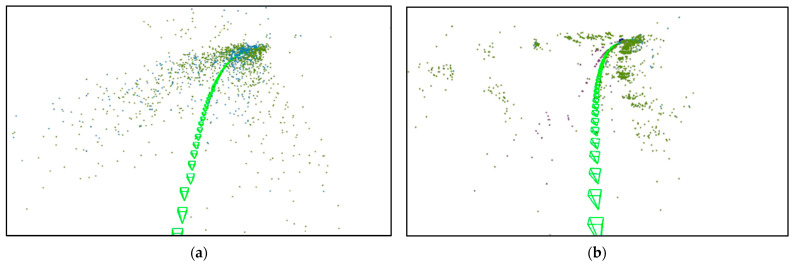

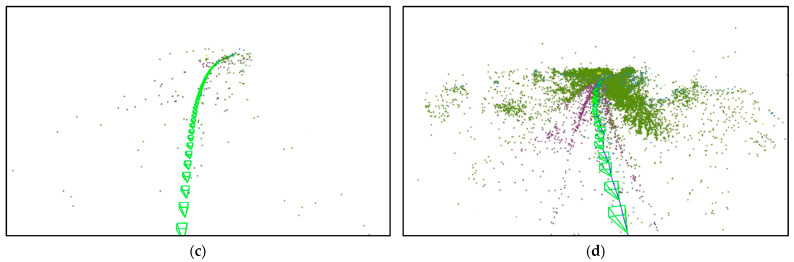
Qualitative analysis of map reload. (**a**) Superpoint [32]; (**b**) alike [40]; (**c**) ORB [35]; (**d**) ours.

Continuing from the previous experiment, we conducted corresponding experiments on map update function. We performed a qualitative analysis using the loop closure sections in red trajectory of the campus dataset. As shown in Figure 11, the scenes during two passes through the same section of the road were compared. The experimental results shown in Figure 12 demonstrate that we effectively implemented the map update function. Figure 13 shows our map reloading function; we use different feature point detection methods to make a qualitative comparison. As we can be seen from Figure 13, our method can effectively capture a large number of feature points, which has a huge improvement in the accuracy and success rate of scene relocation.

### 3.7. Ablation Experiments

To validate the effectiveness of each component in the proposed fusion network of feature point detection and semantic segmentation, we conducted a series of ablation experiments. The experiments were performed on the HPatch dataset [36], with ground truth for semantic segmentation provided by SAM [41]. Evaluation metrics included Repeatability, accuracy, mIoU, and FPS. All experiments used the same training hyperparameters.

- Baseline-A: Feature point detection network only. The semantic segmentation branch is removed, and the feature point detection network is constructed purely with the C2F module.
- Baseline-A1: Similar to Baseline-A, but with reduced Conv4 and C2F_4 layers.
- Baseline-B: Semantic segmentation network only. The feature point detection branch is removed, keeping only the STDC-seg backbone, with output as standard semantic segmentation.
- Naive Fusion: Simple parallel training. C2F and STDC-seg branches run in parallel without any multi-task feature interaction mechanism.
- Proposed Full Model: The full fusion model with shared input head network, and dual-branch architecture of C2F and STDC-seg running in parallel.

As shown in Table 5, when combining the feature point detection and semantic segmentation tasks, our proposed network achieves accuracy comparable to that of the naive combination of the two tasks, but with a significant advantage in speed. The ablation study confirms the rationality of our network design.

## 4. Discussion

From dataset-based evaluations to real-world experiments, this study conducts six distinct experiments to comprehensively validate the effectiveness of the proposed algorithm. Experimental results demonstrate that SGF-SLAM exhibits strong robustness and high stability, achieving outstanding performance in both accuracy and speed. Notably, the SGF-net achieves excellent results with an mIoU of 74.2 on the semantic segmentation task and accuracy (10°) of 75.3 on the feature point matching task, while simultaneously increasing inference speed by 23%. As a lightweight and efficient multi-task network, SGF-net provides robust perceptual capabilities, making it a promising front-end perception module for autonomous driving applications. Moreover, the SGF-SLAM algorithm addresses a key limitation of previous SLAM methods integrated with Gaussian Splatting, which primarily focus on indoor environments. In contrast, SGF-SLAM not only enables real-time localization in road environments, which SfM-based Gaussian Splatting methods lack but also avoids the typical initialization failures associated with SfM. Building upon the Photo-SLAM framework, our approach achieves comparable rendering speed and localization accuracy while improving PSNR by 10%, SSIM by 12%, and reducing LPIPS by 58%. This is mainly due to the dynamic object removal and the Sematic Gaussian Filter method. Additionally, the algorithm effectively removes pedestrians and vehicles from road scenes, reconstructing a clean and visually consistent road environment. This contribution fills a notable gap in the domain of vision-based road scene reconstruction.

However, certain limitations remain. For instance, failure in tracking inevitably leads to degraded reconstruction performance. Although our SLAM-based method achieves a significantly higher localization success rate than SfM-based methods in the tested datasets, it still faces difficulties in scene reconstruction when tracking failures occur. In addition, the most critical limitation lies in memory consumption, which increases rapidly with the scale of scene reconstruction. This issue is especially pronounced on our test platform equipped with a single NVIDIA RTX 3090 Ti GPU (24 GB RAM) (Santa Clara, CA, USA). Although recent efforts have focused on memory compression techniques for Gaussian Splatting, the problem of high memory usage in large-scale road scenes remains a considerable challenge.

Future work will focus on enhancing the perceptual capabilities of the algorithm, improving computational efficiency, and employing more optimized tools to accelerate network inference. Additionally, we plan to integrate memory compression strategies to reduce the memory footprint of the SGF-SLAM system, paving the way for broader deployment in real-world applications.

## 5. Conclusions

This paper presents a SLAM system that combines semantic information expansion, dynamic object masking, feature point detection, and map update techniques. Semantic expansion masks dynamic objects and their reflections, while multi-view ensemble recovers occluded areas. Semantic Gaussian Filter generates clean Gaussian point clouds, improving mapping accuracy and stability. The system provides stable maps under various conditions, enhancing SLAM robustness and accuracy, especially in complex road environments. We validated the system across multiple datasets, showing it effectively suppresses interference, improving accuracy and robustness for autonomous driving and robot localization.

## Figures and Tables

**Table 1:** Quantitative feature matching results on Hpatches datasets [36].

Methods	Acc (5°) ↑	Acc (10°) * ↑	Dim ↓	FPS ↑
ORB [28]	13.8	31.9	256-b	**45.6**
Superpoint [32]	45.0	67.4	256-f	4.3
Xfeat [31]	41.9	74.9	64-f	22.5
seperate ours	**42.1**	75.2	64-f	29.1
ours	42.0	**75.4**	**64-f**	35.8

* The proportion of poses where the maximum angular error is below 10 degrees [31].

**Table 2:** Quantitative stereo tracking results on KITTI odometry dataset [37].

Methods	$t_{abs}$ (m) ↓	$t_{rel}$ (deg/100 m) ↓	$r_{rel}$ (%) ↓
ORB-SLAM3 [28]	2.56	0.22	0.72
Photo-SLAM [13]	2.55	0.24	0.77
DROID-Splat [14]	10.53	1.66	2.32
ours	**2.37**	**0.21**	**0.69**

**Table 3:** Quantitative rendering results on KITTI 360 datasets [4] and VKITTI datasets [39].

Methods	KITTI Scene 02			KITTI Scene 06			vKITTI Scene 02		
	PSNR ↑	SSIM ↑	LPIPS ↓	PSNR ↑	SSIM ↑	LPIPS ↓	PSNR ↑	SSIM ↑	LPIPS ↓
Photo-SLAM [13]	24.04	0.654	0.182	24.67	0.689	0.141	23.92	0.611	0.098
Hugs [22]	25.42	0.821	0.092	28.20	**0.919**	0.027	26.21	0.911	0.040
Ours	26.13	**0.828**	**0.088**	**29.40**	0.906	**0.026**	**27.49**	**0.913**	**0.037**

**Table 4:** Quantitative monocular tracking and mapping results on our campus datasets.

Methods	PSNR ↑	SSIM ↑	LPIPS ↓	Tracking FPS ↑	Rendering FPS ↑	$t_{abs}$ (m) ↓
ORB-SLAM3 [28]	-	-	-	**59.31**	-	2.32
Photo-SLAM [13]	21.33	0.778	0.365	47.47	**798.72**	2.47
HUGs [22]	22.26	0.820	0.184	-	92.57	-
Ours	**23.38**	**0.870**	**0.156**	25.64	782.63	**2.26**

**Table 5:** Quantitative ablation experiments for SGF-net on Hpatches datasets [36].

Methods	Repetability ↑	Acc (10°) * ↑	mIoU ↑	FPS ↑
Baseline-A	71.4	**76.1**	-	37.5
Baseline-A1	45.0	55.7	-	41.3
Baseline-B	-	-	**74.5**	**88.3**
Naive Fusion	70.9	75.6	74.3	29.1
Proposed Full Model	**71.5**	75.3	74.2	35.8

* The proportion of poses where the maximum angular error is below 10 degrees [31].

## Data Availability

The original contributions presented in this study are included in the article. Further inquiries can be directed to the corresponding author.
